# Effects of citrus essential oils on the oxidative stability of microencapsulated fish oil by spray-drying

**DOI:** 10.3389/fnut.2022.978130

**Published:** 2023-01-06

**Authors:** Mustafa Durmus, Yesim Özogul, Gulsun Ozyurt, Yilmaz Ucar, Ali Riza Kosker, Hatice Yazgan, Salam A. Ibrahim, Fatih Özogul

**Affiliations:** ^1^Department of Seafood and Processing Technology, Faculty of Fisheries, Çukurova University, Adana, Turkey; ^2^Fatsa Faculty of Marine Science, Ordu University, Ordu, Turkey; ^3^Department of Food Hygiene and Technology, Faculty of Ceyhan Veterinary Medicine, Çukurova University, Adana, Turkey; ^4^Food Microbiology and Biotechnology Laboratory, North Carolina Agricultural and Technical State University, Greensboro, NC, United States

**Keywords:** fish oil, microencapsulation, spray-drying, lipid oxidation, citrus essential oil

## Abstract

The effects of citrus essential oils (orange, lemon, mandarin, and grapefruit) on the oxidative stability of microencapsulated fish oil by spray-drying were evaluated. The encapsulation efficiency of microcapsules was in the range of 42.25 and 62.43%. Twelve active substances were determined as major volatile components of citrus essential oils. The highest phenolic content was obtained from grapefruit essential oil (44.32 mg GAE/g). Lower values of thiobarbituric acid reactive substances (TBARs) were obtained for microencapsulated fish oils with essential oils compared to control. At the end of storage, the highest peroxide value (PV) was observed in the control group (25.30 meq O_2_/kg oil) while the lowest value was in the lemon (13.40 meq O_2_/kg oil) and orange group (13.91 meq O_2_/kg oil). The results of this study showed that citrus essential oils can be used to improve the oxidative stability of fish oil microcapsules.

## 1. Introduction

Fish oils are very important source of nutrients due to polyunsaturated fatty acids (PUFAs) such as eicosapentaenoic acid (EPA) and docosahexaenoic acid (DHA) in their structure. Researcher reported that the consumption of PUFAs, especially DHA and EPA can prevent the occurrence of many diseases, such as heart arrhythmias, triglyceride concentrations, blood pressure, and platelet aggregation (1). In addition, fish oils represent a functional food ingredient and play a very important role in health (2). For instance, human diets supplemented with fish oils were specified to have beneficial impacts in the prevention of cognitive disorders (3) and on brain development for children and aged individuals (4). Moreover, omega-3 fatty acids protect against the expansion of certain cancers such as breast (5) and prostate cancer (6). While some of the consumers meet the required omega-3 requirements by consuming seafood, for many, direct fish oil purchase has provided a large and important source worldwide, especially for children and pregnant women. However, fish oil has a strong odor and can easily be oxidized as long as it is not protected. The oxidative degradation of unsaturated fatty acids in fish oil results in loss of nutritional value and off-flavors (7). The primary products of lipid oxidation are known as hydroperoxides. These compounds break down into secondary oxidation products such as aldehydes and ketones which cause the unpleasant odors associated with rancid oils. Therefore, antioxidants and different processing technologies are used to protect the quality of fish oils. One of the most important of these technologies is microencapsulation (8).

Microencapsulation is a technology that permits food components or bioactive ingredients (“core” material) to be surrounded with a polymer matrix or “wall.” Thanks to microencapsulated food components, many functional products that are thought to be technically impossible are now producible and can be used as a food ingredient. These ingredients are completely enclosed with a coating material, thereby removing unnecessary features from the original component. Microencapsulation can provide multiple advantages to the food components. For instance, the encapsulated materials are protected against unfavorable reactions such as lipid oxidation and nutritional deterioration throughout production and poor handling, and poor storage conditions (9, 10). Another important property of microencapsulation is that it protects against volatile loss. In light of these information, Shahidi and Han (11) proposed six reasons for the application of microencapsulation in the food industry; to reduce the reactivity of the main component because of the environmental factors, to decrease the rate of transfer of the external component to the main component material, to encourage easier usage, to control the release of the main component material, to mask the taste of the main component and to dilute the main component material when used in a very small amount.

Although many techniques have been developed for microencapsulation of food components, spray-drying is the technique most commonly used in the food industry due to its low cost and flexible process. Spray-drying is a process used to obtain dust by atomizing a liquid product in a stream of hot gas. It is necessary to use high temperatures in this process, but the contact time is too short (a few seconds), that is, energy is used only for evaporation, not for the increase of the temperature of the powder particles. For this reason, it possible to apply this technology to encapsulate heat-sensitive materials with low thermal degradation. Thus, it can also be applied for the encapsulation of bioactive compounds such as omega 3 fatty acids and phytosterols, which are generally heat sensitive. Although microencapsulation is an important technique for stabilization of fish oils, additional stabilization with antioxidants such as essential oils (EOs) is also necessary to provide maximum protection during processing and subsequent storage of the microencapsulated bioactive components (7). Therefore, researchers all over the world are trying to characterize the range of biological properties of EOs including antimicrobial, antioxidant, antiviral, anticancer, anti-inflammatory, antimutagenic, immunomodulator, and antiprotozoal activities (2, 12).

One of the most remarkable EOs groups in recent years is citrus EOs. By-products such as citrus pulp and peels are considered a natural source of flavonoids, which are one of the phenolic compound groups. It is stated that the peel of the fruit that constitutes about half of the citrus fruit contains the highest concentration of flavonoids in the fruit. Citrus peels are reported to be rich in characteristic flavanone glycosides, especially naringin, hesperidin, narirutin, and neohesperidin (13). Gorinstein et al. (14) reported that the total phenolic contents in lemon, orange, and grapefruit peels were found to be quite higher than the edible part of the fruit itself. Citrus essential oils contain mixtures of many components, including terpenes, sesquiterpenes, aldehydes, alcohols, and esters (15). d-Limonene, linalool, γ-terpinene, linalyl acetate, α-terpineol, geranyl acetate, β-pinene and terpinolene, are functioning components of citrus essential oils, and play important roles as antioxidant, anti-inflammatory, antidepressant, anticarcinogenic, and antifungal processes (16). Many essential oils have high antibacterial, antiviral and antifungal activity, thus have an important role as an antiseptic in beverages and food products. Related researches have also shown that essential oils can not only provide flavor to food, but also be the cause of coloring, antioxidant, bacteriostatic and antiseptic activities, which can be applied as important natural preservatives/additives (17–19).

One of the way to reduce oxidation is the microencapsulation process in order to protect PUFAs. This process traps lipid droplets (hydrophobic core material) in a film provided by one or several wall materials, including proteins, starches, polysaccharides, thus delivering a physical barrier between the active material and the environment as well as decreasing contact and reaction with oxygen, light, heat, and metal ions (20). There are many factors that affect the oxidative stability of encapsulated oils such as the presence of antioxidants, oil quality, particle size, oil particle distribution, particle density, surface area, wall materials, storage temperature, moisture content, and water activity (21). In previous studies some wall materials have been used for microencapsulation of fish oil such as butter milk (22), mesquite gum and chitosan (23), konjac glucomannan-soybean protein isolate (24), whey protein isolate and octenylsuccinic anhydride modified starch (25), maltodextrin and sodium caseinate (8).

As far as is known from studies, research of citrus essential oils on the stability of fish oil has never been reported, especially in spray drying conditions. Therefore, in this study, the effects of citrus EOs such as orange, lemon, mandarin, and grapefruit on the oxidative stability of microencapsulated fish oil by spray-drying were investigated.

## 2. Materials and methods

### 2.1. Materials

The raw material of the study, anchovy (*Engraulis encrasicolus*) oil, which is suitable for human consumption, was obtained from a company operating in the province of Trabzon, Türkiye (Kobyalar Group Co.). Sodium caseinate and maltodextrin (Alfasol, Türkiye) were used as coating materials for encapsulation of anchovy oil (Hamasol, Türkiye). Essential oils of grapefruit, mandarin, orange, and lemon peels were obtained from a local company (BIOMESI Bioagrotechnology R&D, Adana, Türkiye).

### 2.2. Methods

#### 2.2.1. Preparation of emulsions

The composition and proportions of the emulsions are shown in Table 1. The emulsion was prepared by dissolving 1:1 sodium caseinate (%86.5 protein) and maltodextrin (DE:18-20) in 55°C water for 1 h and then allowing them outside to cool down. Fish oil was added to this mixture and homogenized in ultra-turrax (IKA T25, Baden-Württemberg, Germany) at 14.000 rpm for 10 min in a cold environment and then the control group was formed with this. After that mandarin, grapefruit, orange, and lemon peel EOs were added in the ratios indicated in Table 1 for the treatment groups and homogenized with ultra-turrax.

**Table 1:** Composition and proportions of emulsions.

**Groups**	**Fish oil (%)**	**Sodium caseinat (%)**	**Maltodextrin (%)**	**Mandarin EOs (%)**	**Grapefruit EOs (%)**	**Orange EOs (%)**	**Lemon EOs (%)**	**Water (%)**
Control (C)	10	10	10	-	-	-	-	70
Mandarin (M)	10	10	10	2.5	-	-	-	67.5
Grapefruit (G)	10	10	10	-	2.5	-	-	67.5
Orange (O)	10	10	10	-	-	2.5	-	67.5
Lemon (L)	10	10	10	-	-	-	2.5	67.5

#### 2.2.2. Spray drying process and storage conditions

The emulsions were dried and pulverized in a laboratory spray dryer equipped with 0.7 mm diameter spray nozzle (Buchi Mini Spray Dryer B-290, Switzerland). The emulsions were fed to the drying chamber by a peristaltic pump using a feed flow rate of 0.5 L/h by continuous homogeneous mixing under the magnetic stirrer before and during drying. Spray dryer (SD) inlet temperature is 160°C and outlet temperature is 90 ± 5°C. The aspirator flow rate is 35 m^3^/h and pump speed is set to 20%. At the end of the process, powdered products were placed in dark glass bottles and stored at room temperature (24 ± 1°C) for 12 weeks. For chemical analyzes, 3 bottles of microencapsulated fish oil for each group were selected randomly on the weeks 0, 2, 4, 5, 6, 7, 8, 9, 10, 11, and 12, and all analyses were carried out at least in triplicate.

#### 2.2.3. Determination of volatile compounds of EOs

The volatile compounds of EOs were determined using a GC-MS (Clarus 500, Perkin Elmer, Waltham, MA, USA) equipped with a silica capillary SGE column (BPX5, 60 m Å~0.25 mm, ID 0.25 um, Perkin Elmer, Shelton, CT, USA). The GC operating settings were documented in detail in a previous study (12, 26).

#### 2.2.4. Determination of total phenolic content in EOs

TPC of each EOs was determined according to the method of Folin-Ciocalteu (27). Concentration for gallic acid standard ranges from 20 to 1,000 μL and results were reported as GA equivalents (mg GAE/g). A total of 11 gallic acid standards were prepared. For the first standard, 1.5 mL Na_2_CO_3_, 0.5 mL Folin Ciocalteu's reagent (Merck 109001), 20 μL gallic acid (Sigma G7384) and 7,980 mL distilled water were added. Later, although Na_2_CO_3_ and Folin Ciocalteu's reagent remained constant in standards, the amount of gallic acid increased and the amount of water decreased. 20 μL of each EOs was dissolved in DMSO (dimethylsulfoxide, Merck, Germany) to yield maximum solubilization and homogenized with the same reagents. Afterwards, the samples were kept in the dark for 2 h at room temperature. The samples were then measured at 760 nm in a spectrophotometer (Shimadzu, UV 1800).

#### 2.2.5. Measurement of microencapsulation efficiency

Each group of microencapsulated fish oil powders (2 g) was transferred to a 250 mL volumetric flask, and 15 mL hexane was added to each sample and the obtained suspension was filtered (using Whatman no. 1 filter paper) followed by rinsing the papers twice with 20 mL hexane (28). The solvent was evaporated using a rotary evaporator (Buchi R210, Rotavapor, Flawil, Switzerland) at 60°C until the volumetric flask reaches a constant weight. The surface oil was designate from the weight differences. Bligh & Dyer extraction method (29) was used for total oil determination. Then, ME was calculated by using following the equation:

*ME* = ⌊(*Total oil*−*Surface oil*)/*Total oil*⌋*x*100

#### 2.2.6. Microcapsule morphology by using scanning electron microscopy

The characterization of surface morphology of microencapsulated fish oils was done using scanning electron microscope (SEM). Microencapsulated fish oil samples were coated with gold for 30 s to a thickness of 2 nm using a Q150R ES Coater (Quorum Technologies, UK). The coated samples were observed using the SEM (Quanta FEG650, USA) at voltage of 20 kV with a magnification of 1,000 and 5,000 and working distance of 10.8–10.9 mm. Carbon tape is used for the adhesion of the material and for conductivity. Morphological structure of microcapsules was characterized by a scanning electron microscope (SEM) in Central Research Laboratory of Cukurova University (CUMERLAB, Adana, Türkiye). The particle sizes of the microencapsulates were also measured using SEM.

#### 2.2.7. Oxidation parameters

The peroxide value (PV) method (30) was performed in order to reveal the PV and free fatty acid (FFA) (30) expressed as percent oleic acid was made by the acidimetric titration after adding ethanol and using phenolphthalein as an indicator. Thiobarbituric acid reactive substances (TBARs) were determined by AOCS method Cd 19–90 (31). TBARs values of the samples were given as mg MA/g of oil.

#### 2.2.8. Statistical analyses

All collected data were analyzed by one-way ANOVA at 5% confidence level using the Duncan multiple range test.

## 3. Results and discussion

### 3.1. Microencapsulation efficiency

Microencapsulation process is important for determining the stability of microcapsules in terms of encapsulation efficiency and surface oil content. Microencapsulation efficiency values of fish oil samples after spray drying are shown in Table 2. The encapsulation efficiency and surface oil content of samples varied from 42.25 to 62.43% and 7.83–12.73% respectively. The highest and lowest microencapsulation efficiency (ME) were calculated for the control (62.43%) and grapefruit (42.25%) groups, respectively. There were not any significant differences between the mandarin and lemon groups (*p* > 0.05), whereas significant differences were observed among other groups (*p* < 0.05). Encapsulation efficiency was higher with the control group, but it was followed by mandarin and lemon. It can be thought that the reason behind the higher encapsulation efficiency of these groups may be due to their higher emulsion viscosity, which aids in faster frying (32) as well as the higher stability of the emulsions (33). In general, the kinetically stable emulsion shows higher encapsulation efficiency, meaning that the particle surfaces of microcapsules have a lower amount of non-encapsulated material (surface oil content). The differences in encapsulation efficiency can also result from the chemical nature of each essential oil group due to a possible chemical interaction with the wall material (34). Yu et al. (35) determined the ME values of fish oil covered with gelatin and arabic scale as 86.90%. Yeşilsu and Özyurt (36) reported that the ME of microencapsulated fish oils prepared with rosemary, thyme, and laurel extracts ranged from 85.62 to 89.40%. In another study, the ME values of microencapsulated fish oil with and without blueberry extract were found to be 53.83–53.86%, respectively (37). In this study, the observed ME values of microencapsulated fish oils in all groups were found to be in accordance with previous studies. The differences observed among our study findings can be attributed to the use of different coating materials, extracts as well as device conditions.

**Table 2:** Microencapsulation efficiency (ME) and surface oil (SO) of microencapsulated fish oil.

	**Control**	**Orange**	**Mandarin**	**Grapefruit**	**Lemon**
ME	62.43 ± 2.72^a^	47.17 ± 0.60^c^	55.06 ± 2.38^b^	42.25 ± 2.13^d^	55.33 ± 3.03^b^
SO	7.83 ± 0.57^d^	12.73 ± 0.87^a^	8.55 ± 0.45^cd^	10.11 ± 0.37^b^	9.45 ± 0.64^bc^

Values represent mean ± SD of 3 replicates in duplicate. Means sharing the same letter in the same line (a–d) are not significantly different (P < 0.05) using Duncan's multiple range test. All units are given in %.

### 3.2. Microcapsule morphology

**Figure 1:**
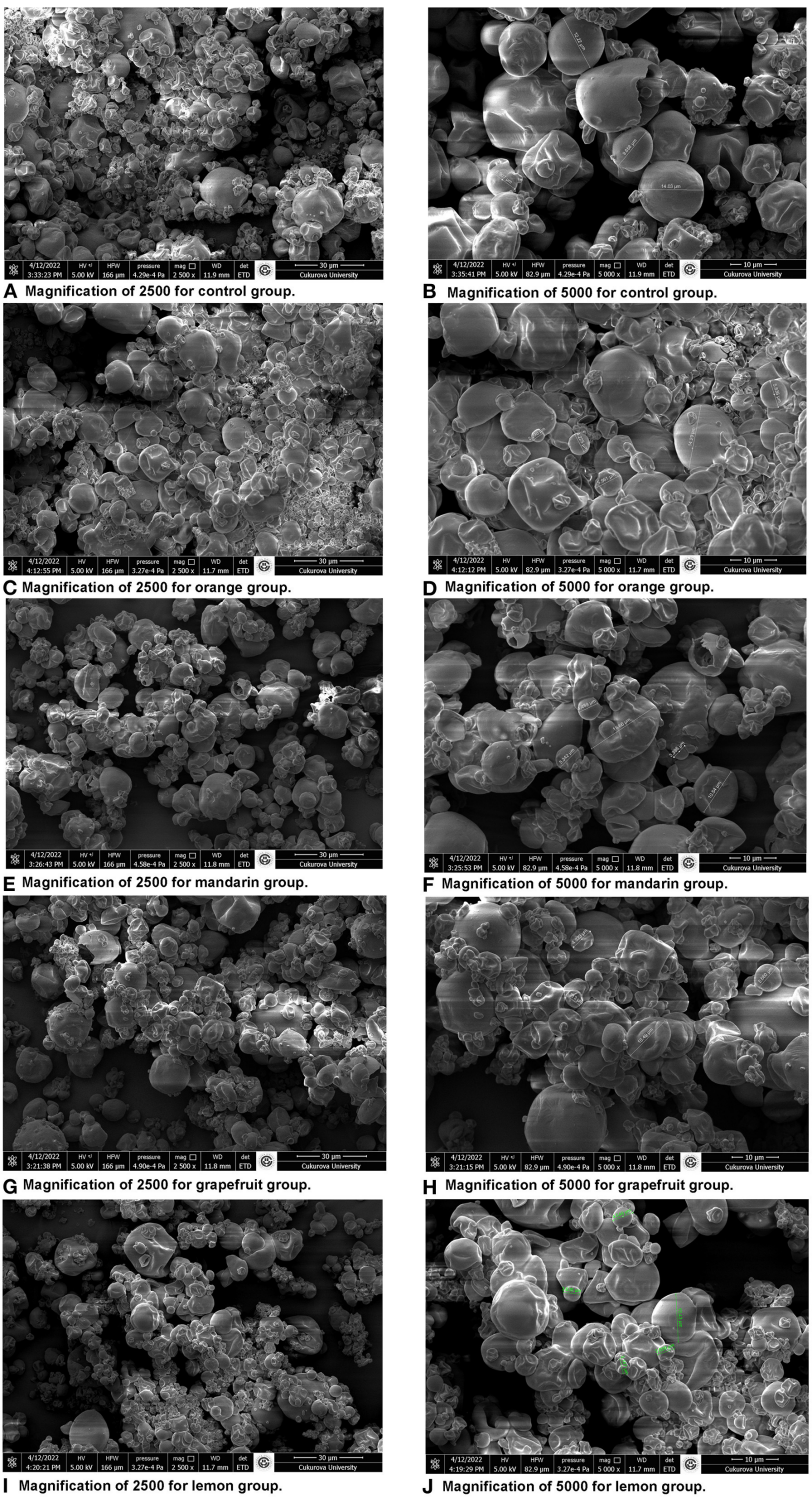
Scanning electron microscopy (SEM) images of the microencapsulated fish oils.

The use of microcapsules in food products depends on the quality, appearance and fluidity of the powder produced. These microcapsule properties are directly dependent on the particle size of the powder (38). Size also plays a role in gastrointestinal performance: microparticles below 800 μm can pass through the pylorus without the effect of gastric emptying, thus eliminating interpersonal and intrinsic (nutritional) differences (39). Figure 1 shows SEM images of microencapsulated fish oil of citrus EOs such as orange, lemon, mandarin and grapefruit. Despite weak clustering in all groups, the overall semblance was homogenous. Microencapsulated fish oils of citrus EOs generally had irregular cylindrical shapes and had little cracking of particles. The roundness of the microcapsules without any cracks or pores is important to protect the core material from oxygen and the release of unwanted oil droplets onto the surface of the particles. Microcapsule morphology varies depending on the coating material, drying conditions, and the level and viscosity of the encapsulated substance (40).

### 3.3. Determination of volatile compounds and total phenolic contents of EOs

The GC-MS analysis showed that 12 active substances (α-Pinene, β-phellandrene, β-pinene, β-nyrcene, cymene, D-limonene, Limonene glycol, Pseudolimonene, Limonene oxide, Bergamotene, β-bisabolene, and Gamma Terpinene) were detected and the highest amount was observed in D-Limonene (Table 3). The highest concentration of D-limonene, a terpene group compound that gives plants their distinctive odor, especially in citrus plant's peels, was observed in grapefruit (92.85)> orange (90.54)> mandarin (87.54)> lemon (49.91), respectively. Unlike other groups, cymene, β-pinene, and α-pinene values were found to be high in lemon EOs. Shah and Mehta (41) investigated the antioxidant activity of d–limonene and found the concentration-dependent on the antioxidant activity of D-Limonene by their antioxidant assays. Similar results were also reported by Piccialli et al. (42). Gülay Kirbaşlar et al. (43) reported that Turkish lemon, grapefruit, bitter and sweet orange, and mandarin peel oils had high levels of monoterpene hydrocarbon (89.9%) and limonene (61.8, 92.5, 94.1, 91.6, and 90.7%, respectively). The level of D-limonene, which is the main component of lemon essential oil, was determined as 52.85% by Yazgan et al. (26) and 46.93% by Moosavy et al. (44). Çoban (45) reported that limonene, one of the main active ingredients in essential oils, is between 71.9 and 88.6% in mandarin and 69.7 in orange. In this study, β-myrcene and β-pinene were identified as having the highest abundance of other components. In another study, limonene was found to be the most common component (95.5%) in the essential oils of 15 different mandarin peels (46). According to German pharmacopeia, limonene in orange peel should be around 90% (Plant Drug Analysis). Orange has been reported to contain more than 170 bioactive compounds (47). Mira et al. (48) reported that the essential oils of orange peels contain 99.5% limonene. These differences can be explained by the fact that the extraction methods used were different as well as the geographies where the samples were taken. In addition to these, parameters such as soil, air, water conditions, sampling times in which plants grow can also cause differences in essential oil compositions.

**Table 3:** Major volatile component of citrus peels essential oils identified by GC/MS.

**Compound name**	**Orange (%)**	**Lemon (%)**	**Grapefruit (%)**	**Mandarin (%)**
α-Pinene	1.57	3.37	1.38	1.67
β-Phellandrene	1.47	1.38	1.33	^*^
β-Pinene	1.15	13.54	^*^	1.05
β-Myrcene	2.48	1.05	3.42	3.33
Cymene	1.14	13.29	^*^	1.69
D-Limonene	90.54	49.91	92.85	87.54
Limonene glycol	^*^	2.31	^*^	^*^
Pseudolimonen	^*^	1.19	^*^	^*^
Limonene oxide	^*^	1.04	^*^	^*^
Bergamotene	^*^	1.52	^*^	^*^
β-Bisabolene	^*^	2.00	^*^	^*^
Gamma terpinene	^*^	^*^	^*^	2.93

^*^Minor compounds of < 1% were not shown (n = 3). All units are given in %.

**Figure 2:**
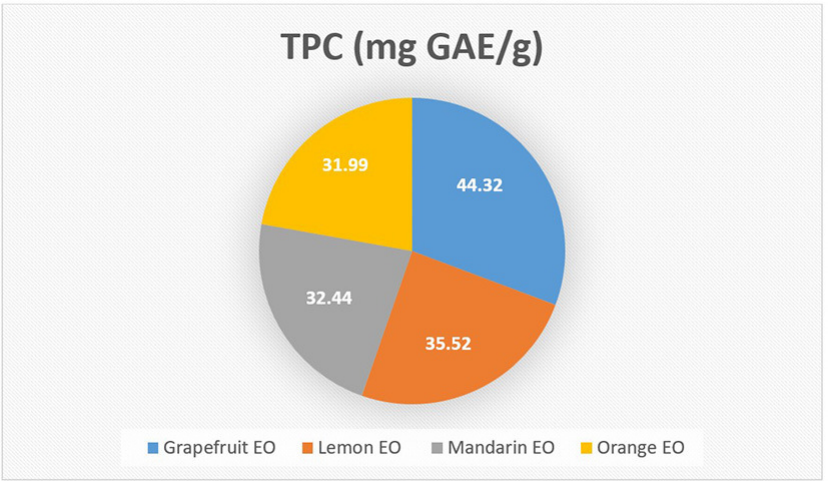
The total phenolic content of essential oils.

The highest TPC was obtained from grapefruit EO (44.32 mg GAE/g), followed by lemon EO (35.52 mg GAE/g), mandarin EO (32.44 mg GAE/g), and orange EO (31.99 mg GAE/g) (Figure 2). Güzel and Akpinar (49) also reported similar results for citrus EOs. In addition, Sir Elkhatim et al. (50) reported that grapefruit peels had the highest total phenolic content, followed by lemons and oranges (77.3, 49.8, and 35.6 mg GAE/g of peels, respectively). Abeysinghe et al. (51) reported that the total phenolic content of *C. unshiu* (Satsuma mandarin), *C. reticulata* (mandarin), and *C. sinensis* (orange) ranged from 18.5 to 38.5% and determined that the main phenolic component was hesperid.

### 3.4. Changes in oxidative stability

Microencapsulation by spray drying is recognized as an important technique for heat sensitive materials due to its relatively short drying time, low cost and easy application for industrial scale production (52). In this process, it is of great importance to control the internal temperature, feed rate, flow rate and feed temperature in the dryer and to provide that the temperature of atomized particle doesn't exceed 100°C. In microencapsulation by spray drying, it is thought that the high temperatures applied do not harm the product, because the short drying time and the theoretical wet bulb temperature (30–50°C) are not exceeded. However, it should be noted that the high inlet temperatures applied in the process may adversely affect the heat-sensitive components. Encina et al. (53) reported that the inlet temperatures applied in fish oil microencapsulation by spray drying were between 120°C and 210°C. Some researchers have reported that increasing the inlet air temperature in spray drying increases oxidative reactions in fish oils (53, 54). However, a low inlet temperature was reported to affect the morphology of fish oil microparticles and cause agglomeration and particle precipitation as a result of high moisture content (54, 55). Our previous study showed that the inlet temperature of 160°C, as applied in this study, can be preferred to overcome these problems (54).

TBARs represent malondialdehydes, which are secondary degradation products of lipid oxidation, and are one of the criteria showing the rancidity of oils. TBARs values of microencapsulated fish oils with citrus peel EOs are given in Table 4. At the beginning of the study, the TBARs value of the control group was determined as 2.03 mg MA/kg. At the beginning of storage, the lowest TBARs value was determined in the mandarin group (0.88 mg MA/kg) while the highest TBARs value was determined in the lemon group (1.73 mg MA/kg) among the groups containing essential oil. Fluctuations in values were observed in all groups during the storage period. This could be attributed to the other substances present in foods, such as sugars, acids, esters, amino acids and oxidized proteins, which may also react with TBA. The groups with the highest TBARs values were observed in the lemon (1.94 mg MA/kg), mandarin (1.93 mg MA/kg), and grapefruit (1.72 mg MA/kg) groups at the 2^nd^ week of storage. However the highest value in the orange (1.86 mg MA/kg) group was observed at the 8^th^ week of storage. At the end of storage, the TBARs values of fish oils with citrus EOs were significantly lower than those of the control group (*P* < 0.05). Among the citrus groups, the lowest TBARs value was determined in the lemon group (0.29 mg MA/kg). Li et al. (37) determined the TBARs value of spray dried microcapsules with blueberry extract as 0.487 mg MA/kg. Solval et al. (56) reported that the TBARs values that they could not detect in the crude oil ranged from 0.11 to 0.12 mg MA/kg fat after microencapsulation by spray drying. Özyurt et al. (57) reported that the TBA values of fish oils microencapsulated by spray drying were in the range of 0.8–1.68 mg MA/kg throughout storage period, which were lower than the acceptability limit of 2 mg MA/kg according to Connell (58). Binsi et al. (59) reported that the TBARs values of fish oil microencapsulated by spray drying samples with sage polyphenol exceeded the limit after 24 h when the samples were kept at 60°C. It has been reported that the end product is unacceptable when 1.37 g MA/kg is exceeded in fish oil (60). In the current study, although TBARs values showed fluctuations in all groups during storage period, their levels did not exceed 2.03 mg MA/kg. As a result, it has been determined that citrus peel EOs are a good oxidation retarder for microencapsulation of fish oils.

**Table 4:** The changes in PV, TBARs, and FFA contents of microencapsulated fish oil during storage.

**Storage days (weeks)**	**Control**	**Orange**	**Mandarin**	**Grapefruit**	**Lemon**
**TBARs content (mg malonaldehyde (MA)/kg oil)**					
0	2.03 ± 0.07^a^	1.68 ± 0.07^b^	0.88 ± 0.07^d^	1.36 ± 0.03^c^	1.73 ± 0.04^b^
2	1.92 ± 0.04^a^	1.51 ± 0.07^c^	1.93 ± 0.04^a^	1.72 ± 0.06^b^	1.94 ± 0.22^a^
4	1.75 ± 0.05^a^	0.81 ± 0.06^c^	1.48 ± 0.06^b^	0.85 ± 0.03^c^	0.63 ± 0.03^d^
5	1.84 ± 0.06^a^	1.60 ± 0.13^b^	0.94 ± 0.02^d^	1.39 ± 0.07^c^	1.02 ± 0.05^d^
6	1.82 ± 0.06^a^	1.71 ± 0.06^b^	0.56 ± 0.03^c^	1.71 ± 0.07^b^	0.38 ± 0.01^d^
7	1.01 ± 0.13^c^	0.56 ± 0.02^d^	1.14 ± 0.05^b^	0.45 ± 0.03^e^	1.89 ± 0.08^a^
8	0.78 ± 0.05^b^	1.86 ± 0.06^a^	0.51 ± 0.01^d^	0.65 ± 0.04^c^	0.63 ± 0.05^c^
9	0.58 ± 0.06^d^	0.77 ± 0.02^b^	1.10 ± 0.06^a^	0.68 ± 0.04^c^	0.56 ± 0.02^d^
10	1.38 ± 0.07^a^	1.23 ± 0.04^b^	0.73 ± 0.06^c^	1.40 ± 0.04^a^	1.29 ± 0.04^b^
11	0.95 ± 0.05^b^	1.34 ± 0.02^a^	0.40 ± 0.05^c^	1.34 ± 0.04^a^	0.25 ± 0.02^d^
12	0.90 ± 0.03^a^	0.74 ± 0.01^b^	0.62 ± 0.06^c^	0.75 ± 0.02^b^	0.29 ± 0.01^d^
**FFA content (% FFA as oleic acid)**					
0	3.96 ± 0.07^e^	4.74 ± 0.02^c^	4.46 ± 0.07^d^	6.36 ± 0.00^a^	5.11 ± 0.01^b^
2	15.81 ± 0.13^a^	13.72 ± 0.73^b^	8.75 ± 0.01^d^	14.56 ± 0.86^ab^	10.43 ± 0.04^c^
4	7.96 ± 0.09^c^	13.24 ± 0.06^a^	7.21 ± 0.06^d^	12.62 ± 0.26^b^	13.10 ± 0.13^a^
5	8.03 ± 0.04^c^	7.23 ± 0.02^d^	14.66 ± 0.01^a^	6.34 ± 0.10^e^	9.16 ± 0.06^b^
6	9.39 ± 0.03^c^	4.81 ± 0.01^e^	12.28 ± 0.87^a^	6.35 ± 0.02^d^	10.54 ± 0.07^b^
7	9.37 ± 0.04^b^	8.46 ± 0.03^c^	7.32 ± 0.08^d^	11.10 ± 0.06^a^	6.59 ± 0.04^e^
8	12.59 ± 0.85^a^	5.99 ± 0.01^d^	11.44 ± 0.24^b^	11.08 ± 0.18^b^	8.92 ± 0.30^c^
9	10.23 ± 0.11^a^	6.87 ± 0.45^d^	5.81 ± 0.11^e^	7.66 ± 0.11^c^	9.26 ± 0.02^b^
10	9.37 ± 0.05^b^	7.80 ± 0.86^c^	11.68 ± 0.12^a^	7.93 ± 0.09^c^	7.89 ± 0.10^c^
11	13.18 ± 0.07^a^	6.01 ± 0.04^c^	13.21 ± 0.04^a^	7.91 ± 0.10^b^	12.34 ± 0.76^a^
12	9.98 ± 0.76^b^	7.19 ± 0.07^c^	10.22 ± 0.03^b^	7.89 ± 0.14^c^	12.45 ± 0.89^a^
**PV values (meq O2/kg)**					
0	11.21 ± 0.28^c^	11.19 ± 1.05^c^	13.09 ± 0.53^b^	14.41 ± 0.65^a^	12.35 ± 0.62^bc^
2	14.75 ± 1.01^ab^	12.86 ± 0.86^b^	9.93 ± 0.58^c^	16.95 ± 0.86^a^	12.76 ± 2.24^b^
4	17.11 ± 0.14^a^	12.35 ± 0.77^c^	14.43 ± 0.08^b^	11.30 ± 0.09^d^	10.31 ± 0.13^e^
5	23.78 ± 1.99^a^	13.45 ± 0.44^bc^	14.95 ± 1.17^b^	12.43 ± 1.53^c^	14.66 ± 1.07^bc^
6	21.21 ± 0.79^a^	12.56 ± 0.92^b^	13.84 ± 0.74^b^	13.56 ± 1.24^b^	10.72 ± 0.57^c^
7	20.51 ± 1.56^a^	11.70 ± 1.26^c^	18.21 ± 2.20^a^	15.47 ± 1.66^b^	13.14 ± 0.13^bc^
8	16.24 ± 1.27^a^	12.57 ± 0.45^b^	10.41 ± 0.01^c^	12.73 ± 0.63^b^	12.77 ± 1.38^b^
9	14.54 ± 1.07^a^	9.43 ± 0.96^bc^	8.17 ± 0.09^c^	10.48 ± 0.73^b^	10.43 ± 0.54^b^
10	22.85 ± 0.81^a^	10.30 ± 1.18^c^	10.38 ± 1.36^c^	11.95 ± 0.75^b^	12.18 ± 0.87^b^
11	26.89 ± 2.87^a^	17.64 ± 1.09^c^	11.96 ± 0.47^d^	21.17 ± 2.08^b^	13.97 ± 0.87^d^
12	25.30 ± 1.19^a^	13.91 ± 0.27^d^	15.66 ± 0.98^c^	18.00 ± 1.23^b^	13.40 ± 0.58^d^

Values represent mean ± SD of 3 replicates in duplicate. Means sharing the same letter in the same line (a–d) are not significantly different (P < 0.05) using Duncan's multiple range test.

Free fatty acids (FFA) are known to be the result of enzymatic hydrolysis of esterified lipids (61). It is known that there is a relationship between the amount of FFA and fish freshness depending on the storage period. Moreover, as the amount of FFA increases, they can adversely affect the taste of the oil and increase rancidity. Therefore, monitoring changes in FFA content is an important parameter for lipid hydrolysis in fish oils. While the FFA values at the beginning of the storage period were in the range of 3.96–6.36% in all groups, the values were in the range of 7.19–12.45% at the end of storage (Table 4). Fluctuations were observed in the FFA values of microencapsulated fish oils in all groups during storage. The highest values of FFA were observed at the 2^nd^ week of storage for the control, orange, and grapefruit groups (15.81, 13.72, and 14.56% respectively), but at the 4^th^ week for lemon (13.10%) and at the 5^th^ week for mandarin (14.66%). Bimbo (62) reported that the FFA content in raw fish is normally between 1 and 7%. Skelbaek and Andersen (63) reported that the FFA content of microencapsulated oils or fats was below 5%. In this study, it was determined that FFA values at the beginning of storage were close to this recommended limit. However, it was observed that FFA values exceeded the recommended limit toward to end of the storage period.

The formation of peroxides is considered indicative of the first stage of lipid oxidation. The peroxide values (PV) provide information about the degree of oxidation of the oil. The changes in PV of the samples are given in Table 4. Statistical differences were observed in PV among all groups during the storage period (*p* < 0.05). Immediately after microencapsulation, PV changed between 11.19 and 14.41 meq O_2_/kg oil in all groups. Among the treatment groups, the highest PV was observed at week 5 for orange group (13.45 meq O_2_/kg oil), week 7 for mandarin group (18.21 meq O_2_/kg oil), week 11 for grapefruit group (21.17 meq O_2_/kg oil), and week 5 for lemon group (14.66 meqO_2_/kg oil). At the end of storage, the highest PV among the treatment groups was obtained from the grapefruit group (18.00 meq O_2_/kg oil) while the lowest PV was determined in the lemon group (13.40 meq O_2_/kg oil). However, the highest PVs were generally observed in the control group during storage. Yeşilsu and Özyurt (36) reported that the PV of fish oil microencapsulated by spray drying with rosemary, thyme, and laurel extracts were lower than those of the control group containing commercial antioxidants. The researchers found that the addition of rosemary extract delays the formation of peroxide in microencapsulated anchovy oils, and the addition of 1,500 ppm rosemary provides better protection in terms of preventing the formation of peroxide from 250 ppm of BHT. Bakry et al. (64) investigated tuna oil microencapsulated by spray drying stored at 45°C for 28 days and reported that the PV of the group added peppermint oil was two times less than that the group without it. Kolanowski et al. (65) reported that the peroxide values of fish oil microencapsulated by spray drying without antioxidants exceeded 60 mEq during 36 days of storage at room temperature. Hogan et al. (9) reported that PV of fish oil samples microencapsulated by spray drying stored at room temperature for 28 days increased to 85.6 mEq O_2_/kg oil. Compared to these reports, it is seen that the results obtained from the present study are quite low in terms of PV.

Citrus essential oils has increasingly gained the interest as a potential natural antimicrobial and antioxidant agent (66). Although the highest level of phenolic component was found in grapefruit, their contents in other citrus peel EOs tested were similar and comparable to that of grapefruit in the present study. The phenolic compounds of citrus peel oils may correlate with the antioxidant activity of the EOs. The relation between total phenol content and antioxidant activity has been widely studied in different fruit and vegetables (67–69), exhibiting that the free radical scavenging capacity of fruits or vegetables significantly rises with a high level of total polyphenol content (70). Moreover, the phenolic compounds have the capacity to scavenge free radicals by giving a hydrogen atom from their phenolic hygrogen groups (44). As found in this work, citrus essential oils, especially lemon and orange peel EOs were able to reduce oxidation parameters, when compared to the control, showing a good antioxidant capacity. The antioxidant activity of the tested EO might be related to the presence of monoterpenes, particularly limonene and γ-terpinene, which are the major composites of EO and reported to have a good antioxidant activity (71).

## 4. Conclusion

The results of the present study suggest that it may be advisable to add citrus essential oils in fish oil prior to spray drying to protect the highly sensitive omega-3 fatty acids from lipid oxidation. Considering the oxidation parameters obtained, it was determined that microencapsulated fish oils with added citrus essential oils gave better results than the control group. On the other hand, it can be said that the orange and lemon groups are among the most effective groups, especially in terms of PV values, among citrus groups at the end of storage. In addition, the general consumer appreciation of the citrus flavor and odor contributes to masking odor of fishy oil so that people can get enough EPA and DHA in their daily diets.

## Data availability statement

The original contributions presented in the study are included in the article/supplementary material, further inquiries can be directed to the corresponding author.

## Author contributions

MD: original draft preparation, conceptualization, and writing and editing. YÖ: writing and review and editing. GO and YU: conceptualization, software, formal analysis, and writing–original draft. AK and HY: software, formal analysis, and writing–original draft. SI: review and editing and funding. FÖ: review and editing. All authors contributed to the article and approved the submitted version.

## References

[1] Raatz SK Bibus DM. Fish and Fish Oil in Health and Disease Prevention. Elsevier (2016). p. 1–366.

[2] Durmus M. The effects of nanoemulsions based on citrus essential oils (orange, mandarin, grapefruit, and lemon) on the shelf life of rainbow trout (Oncorhynchus mykiss) fillets at 4 ± 2°C. J Food Saf. (2020) 40:1–10. 10.1111/jfs.12718

[3] Graciano MF Leonelli M Curi R Carpinelli AR. Omega-3 fatty acids control productions of superoxide and nitrogen oxide and insulin content in INS-1E cells. J Physiol Biochem. (2016) 72:699–710. 10.1007/s13105-016-0509-1 27474043

[4] He Y Li J Kodali S Chen B Guo Z. Rationale behind the near-ideal catalysis of Candida antarctica lipase A (CAL-A) for highly concentrating ω-3 polyunsaturated fatty acids into monoacylglycerols. Food Chem. (2017) 219:230–9. 10.1016/j.foodchem.2016.09.149 27765222

[5] Rendeiro C Sheriff A Bhattacharya TK Gogola JV Baxter JH Chen H. Long-lasting impairments in adult neurogenesis, spatial learning and memory from a standard chemotherapy regimen used to treat breast cancer. Behav Brain Res. (2016) 315:10–22. 10.1016/j.bbr.2016.07.043 27478140

[6] Itsiopoulos C Hodge A Kaimakamis M. Can the Mediterranean diet prevent prostate cancer? Mol Nutr Food Res. (2009) 53:227–39. 10.1002/mnfr.200800207 19051189

[7] Jeyakumari A Zynudheen AA Parvathy U Binsi PK. Impact of chitosan and oregano extract on the physicochemical properties of microencapsulated fish oil stored at different temperature. Int J Food Prop. (2018) 21:942–55. 10.1080/10942912.2018.1466319

[8] Özyurt G Sakarya Y Durmuş M. Chemical and physical characterization of spray dried fish oil with different combination ratios of wall component. J Food Process Preserv. (In Press). 10.1111/jfpp.17223

[9] Hogan SA O'Riordan ED O'Sullivan M. Microencapsulation and oxidative stability of spray-dried fish oil emulsions. J Microencapsul. (2003) 20:675–88. 10.3109/02652040309178355 12909550

[10] Chen Q McGillivray D Wen J Zhong F Quek SY. Co-encapsulation of fish oil with phytosterol esters and limonene by milk proteins. J Food Eng. (2013) 117:505–12. 10.1016/j.jfoodeng.2013.01.011

[11] Shahidi F Han XQ. Encapsulation of food ingredients. Crit Rev Food Sci Nutr. (1993) 33:501–47. 10.1080/10408399309527645 8216812

[12] Ozogul Y Yuvka I Ucar Y Durmus M Kösker AR Öz M. Evaluation of effects of nanoemulsion based on herb essential oils (rosemary, laurel, thyme and sage) on sensory, chemical and microbiological quality of rainbow trout (Oncorhynchus mykiss) fillets during ice storage. LWT. (2017) 75:677–84. 10.1016/j.lwt.2016.10.009

[13] Khan MK Zill-E-Huma Dangles O. A comprehensive review on flavanones, the major citrus polyphenols. J Food Compos Anal. (2014) 33:85–104. 10.1016/j.jfca.2013.11.00435119637

[14] Gorinstein S Martín-Belloso O Park YS Haruenkit R Lojek A ÍŽ M. Comparison of some biochemical characteristics of different citrus fruits. Food Chem. (2001) 74:309–15. 10.1016/S0308-8146(01)00157-1

[15] Lin LY Chuang CH Chen HC Yang KM. Lime (Citrus aurantifolia (Christm.) swingle) essential oils: Volatile compounds, antioxidant capacity, and hypolipidemic effect. Foods. (2019) 8:398. 10.3390/foods8090398 31500259 PMC6770194

[16] Dosoky NS Setzer WN. Biological activities and safety of citrus spp. Essential oils Int J Mol Sci. (2018) 19:1–25. 10.3390/ijms19071966 29976894 PMC6073409

[17] Raut JS Karuppayil SM. A status review on the medicinal properties of essential oils. Ind Crops Prod. (2014) 62:250–64. 10.1016/j.indcrop.2014.05.05535405814

[18] Ju J Xu X Xie Y Guo Y Cheng Y Qian H. Inhibitory effects of cinnamon and clove essential oils on mold growth on baked foods. Food Chem. (2018) 240:850–5. 10.1016/j.foodchem.2017.07.120 28946351

[19] Saeed K Pasha I Jahangir Chughtai MF Ali Z Bukhari H Zuhair M. Application of essential oils in food industry: challenges and innovation. J Essent Oil Res. (2022) 34:97–110. 10.1080/10412905.2022.2029776

[20] Jiménez-Martín E Gharsallaoui A Pérez-Palacios T Carrascal JR Rojas TA. Suitability of using monolayered and multilayered emulsions for microencapsulation of ω-3 fatty acids by spray drying: effect of storage at different temperatures. Food Bioprocess Technol. (2015) 8:100–11. 10.1007/s11947-014-1382-y

[21] Ghnimi S Budilarto E Kamal-Eldin A. The new paradigm for lipid oxidation and insights to microencapsulation of omega-3 fatty acids. Compr Rev Food Sci Food Saf. (2017) 16:1206–18. 10.1111/1541-4337.12300 33371591

[22] Zhang Y Pang X Zhang S Liu L Ma C Lu J. Buttermilk as a wall material for microencapsulation of omega-3 oils by spray drying. LWT. (2020) 127:109320. 10.1016/j.lwt.2020.109320

[23] Espinosa-Andrews H Morales-Hernández N García-Márquez E Rodríguez-Rodríguez R. Development of fish oil microcapsules by spray drying using mesquite gum and chitosan as wall materials: physicochemical properties, microstructure, and lipid hydroperoxide concentration. Int J Polym Mater Polym Biomater. (2022) 71:1–10. 10.1080/00914037.2022.2042289

[24] Cui T Chen C Jia A Li D Shi Y Zhang M. Characterization and human microfold cell assay of fish oil microcapsules: Effect of spray drying and freeze-drying using konjac glucomannan (KGM)-soybean protein isolate (SPI) as wall materials. J Funct Foods. (2021) 83:104542. 10.1016/j.jff.2021.104542

[25] Zhu Y Peng Y Wen J Quek SY. A comparison of microfluidic-jet spray drying, two-fluid nozzle spray drying, and freeze-drying for co-encapsulating β-carotene, lutein, zeaxanthin, and fish oil. Foods. (2021) 10:1522. 10.3390/foods10071522 34359390 PMC8303781

[26] Yazgan H Ozogul Y Kuley E. Antimicrobial influence of nanoemulsified lemon essential oil and pure lemon essential oil on food-borne pathogens and fish spoilage bacteria. Int J Food Microbiol. (2019) 306:108266. 10.1016/j.ijfoodmicro.2019.108266 31319195

[27] Gamez-Meza N Noriega-Rodriguez JA Medina-Juarez LA Ortega-Garcia J Cazarez-Casanova R Angulo-Guerrero O. Antioxidant activity in soybean oil of extracts from Thompson grape bagasse. JAOCS. (1999) 76:1445–7. 10.1007/s11746-999-0182-4

[28] Bae EK Lee SJ. Microencapsulation of avocado oil by spray drying using whey protein and maltodextrin. J Microencapsul. (2008) 25:549–60. 10.1080/02652040802075682 18465295

[29] Bligh EG Dyer WJ. A rapid method of total lipid extraction and purification. Can J Biochem Physiol. (1959) 37:911–7. 13671378 10.1139/o59-099

[30] American Oil Chemists Society. Official Methods and Recommended Practices of the American Oil Chemists' Society. Washington, DC: American Oil Chemists Society (1997).

[31] American Oil Chemists Society. Official Methods and Recommended Practices of the American Oil Chemists' Society. Washington, DC: American Oil Chemists Society (1998).

[32] Premi M Sharma HK. Effect of different combinations of maltodextrin, gum arabic and whey protein concentrate on the encapsulation behavior and oxidative stability of spray dried drumstick (Moringa oleifera) oil. Int J Biol Macromol. (2017) 105:1232–40. 10.1016/j.ijbiomac.2017.07.160 28757420

[33] Barbosa MIMJ Borsarelli CD Mercadante AZ. Light stability of spray-dried bixin encapsulated with different edible polysaccharide preparations. Food Res Int. (2005) 38:989–94. 10.1016/j.foodres.2005.02.018

[34] Silva LS Mar JM Azevedo SG Rabelo MS Bezerra JA Campelo PH. Encapsulation of Piper aduncum and Piper hispidinervum essential oils in gelatin nanoparticles: a possible sustainable control tool of Aedes aegypti, Tetranychus urticae and Cerataphis lataniae. J Sci Food Agric. (2019) 99:685–95. 10.1002/jsfa.9233 29971785

[35] Yu F Li Z Zhang T Wei Y Xue Y Xue C. Influence of encapsulation techniques on the structure, physical properties, and thermal stability of fish oil microcapsules by spray drying. J Food Process Eng. (2017) 40:e12576. 10.1111/jfpe.12576

[36] Yeşilsu AF Özyurt G. Oxidative stability of microencapsulated fish oil with rosemary, thyme and laurel extracts: a kinetic assessment. J Food Eng. (2019) 240:171–82. 10.1016/j.jfoodeng.2018.07.021

[37] Li J Solval KM Alfaro L Zhang J Chotiko A Delgado JLB. Effect of blueberry extract from blueberry pomace on the microencapsulated fish oil. J Food Process Preserv. (2015) 39:199–206. 10.1111/jfpp.12222

[38] Mahdi AA Mohammed JK Al-Ansi W Ghaleb ADS Al-Maqtari QA Ma M. Microencapsulation of fingered citron extract with gum arabic, modified starch, whey protein, and maltodextrin using spray drying. Int J Biol Macromol. (2020) 152:1125–34. 10.1016/j.ijbiomac.2019.10.201 31751737

[39] Lengyel M Kállai-Szabó N Antal V Laki AJ Antal I. Microparticles, microspheres, and microcapsules for advanced drug delivery. Sci Pharm. (2019) 87:20. 10.3390/scipharm87030020

[40] Wyspiańska D Kucharska AZ Sokół-Łetowska A Kolniak-Ostek J. Effect of microencapsulation on concentration of isoflavones during simulated in vitro digestion of isotonic drink. Food Sci Nutr. (2019) 7:805–16. 10.1002/fsn3.929 30847160 PMC6392822

[41] Shah BB Mehta AA. In vitro evaluation of antioxidant activity of D-Limonene. Asian J Pharm Pharmacol. (2018) 4:883–7. 10.31024/ajpp.2018.4.6.25

[42] Piccialli I Tedeschi V Caputo L Amato G De Martino L De Feo V. The antioxidant activity of limonene counteracts neurotoxicity triggered byaβ1-42 oligomers in primary cortical neurons. Antioxidants. (2021) 10:937. 10.3390/antiox10060937 34207788 PMC8227170

[43] Gülay Kirbaşlar F Tavman A Dülger B Türker G. Antimicrobial activity of Turkish Citrus peel oils. Pakistan J Bot. (2009) 41:3207–12.

[44] Moosavy MH Hassanzadeh P Mohammadzadeh E Mahmoudi R Khatibi SA Mardani K. Antioxidant and antimicrobial activities of essential oil of lemon (Citrus limon) peel in vitro and in a food model. J Food Qual Hazards Control. (2017) 4:42–8.

[45] Çoban T. Comparison of citrus essential oil components obtained by different methods (Master's Thesis). Çukurova University, Adana, Turkey (2009). p. 57.

[46] Lota ML De Rocca Serra D Tomi F Casanova J. Chemical variability of peel and leaf essential oils of 15 species of mandarins. Biochem Syst Ecol. (2001) 29:77–104. 10.1016/S0305-1978(00)00029-6 11068126

[47] Wang YC Chuang YC Ku YH. Quantitation of bioactive compounds in citrus fruits cultivated in Taiwan. Food Chem. (2007) 102:1163–71. 10.1016/j.foodchem.2006.06.057

[48] Mira B Blasco M Berna A Subirats S. Supercritical CO2 extraction of essential oil from orange peel. Effect of operation conditions on the extract composition. J Supercrit Fluids. (1999) 14:95–104. 10.1016/S0896-8446(98)00111-9

[49] Guzel M Akpinar Ö. Turunçgil Kabuklarinin Biyoaktif Bileşenleri ve Antioksidan Aktivitelerinin Belirlenmesi Determination of Bioactive Compounds and Antioxidant Activities of Citrus Peels. Gümüşhane Üniversitesi Fen Bilim Derg. (2017) 7:153–67.

[50] Sir Elkhatim KA Elagib RAA Hassan AB. Content of phenolic compounds and vitamin C and antioxidant activity in wasted parts of Sudanese citrus fruits. Food Sci Nutr. (2018) 6:1214–9. 10.1002/fsn3.660 30065822 PMC6060895

[51] Abeysinghe DC Li X Sun C De Zhang WS Zhou CH Chen KS. Bioactive compounds and antioxidant capacities in different edible tissues of citrus fruit of four species. Food Chem. (2007) 104:1338–44. 10.1016/j.foodchem.2007.01.047

[52] Zhu P Patel K Lin S Méjean S Blanchard E Chen XD. Simulating industrial spray-drying operations using a reaction engineering approach and a modified desorption method. Dry Technol. (2011) 29:419–28. 10.1080/07373937.2010.501928

[53] Encina C Vergara C Giménez B Oyarzún-Ampuero F Robert P. Conventional spray-drying and future trends for the microencapsulation of fish oil. Trends Food Sci Technol. (2016) 56:46–60. 10.1016/j.tifs.2016.07.014

[54] Özyurt G Durmuş M Özkütük AS Uçar Y. Microencapsulation of fish oil with olive leaf extract instead of synthetic antioxidant and its effects on nutraceutical properties of fish oil at different inlet temperatures. Biomass Convers Biorefinery. (2022) 1:3. 10.1007/s13399-022-03231-4

[55] Wang R Tian Z Chen L. A novel process for microencapsulation of fish oil with barley protein. Food Res Int. (2011) 44:2735–41. 10.1016/j.foodres.2011.06.013

[56] Solval KMS. Spray drying technology for the production and processing of microencapsulated omega-3 fish oil with egg powder (Master's Thesis). Louisiana State University, Baton Rouge, LA, United States (2011). 10.31390/gradschool_theses.1390

[57] Özyurt G Durmuş M Uçar Y Özogul Y. The potential use of recovered fish protein as wall material for microencapsulated anchovy oil. LWT. (2020) 129:109554. 10.1016/j.lwt.2020.109554

[58] Connell JJ. Methods of Assessing Selecting for Quality. 3rd ed. Oxford: Fishing News Books (1990). p. 256. Available online at: https://www.wiley.com/en-in/Control+of+Fish+Quality%2C+4th+Edition-p-9780852382264 (accessed December 20, 2022).

[59] Binsi PK Nayak N Sarkar PC Jeyakumari A Muhamed Ashraf P Ninan G. Structural and oxidative stabilization of spray dried fish oil microencapsulates with gum arabic and sage polyphenols: characterization and release kinetics. Food Chem. (2017) 219:158–68. 10.1016/j.foodchem.2016.09.126 27765212

[60] Kaitaranta JK. Control of lipid oxidation in fish oil with various antioxidative compounds. J Am Oil Chem Soc. (1992) 69:810–3. 10.1007/BF02635921

[61] Pacheco-Aguilar R Lugo-Sánchez ME Robles-Burgueño MR. Postmortem biochemical and functional characteristic of monterey sardine muscle stored at 0°C. J Food Sci. (2000) 65:40–7. 10.1111/j.1365-2621.2000.tb15953.x

[62] Bimbo AP. Guidelines for characterizing food-grade fish oil. Inf Int News Fats, Oils Relat Mater. (1998) 9:473–83.

[63] Skelbaek T Andersen S. A microencapsulated oil or fat product. U.S. Patent No. 6,444,242. Washington, DC: U.S. Patent and Trademark Office (1994).

[64] Bakry AM Fang Z Ni Y Cheng H Chen YQ Liang L. Stability of tuna oil and tuna oil/peppermint oil blend microencapsulated using whey protein isolate in combination with carboxymethyl cellulose or pullulan. Food Hydrocoll. (2016) 60:559–71. 10.1016/j.foodhyd.2016.04.026

[65] Kolanowski W Laufenberg G Kunz B. Fish oil stabilisation by microencapsulation with modified cellulose. Int J Food Sci Nutr. (2004) 55:333–43. 10.1080/09637480410001725157 15369987

[66] Raspo MA Vignola MB Andreatta AE Juliani HR. Antioxidant and antimicrobial activities of citrus essential oils from Argentina and the United States. Food Biosci. (2020) 36:100651. 10.1016/j.fbio.2020.100651

[67] Lutz M Hernández J Henríquez C. Phenolic content and antioxidant capacity in fresh and dry fruits and vegetables grown in Chile. CYTA J Food. (2015) 13:541–7. 10.1080/19476337.2015.1012743

[68] Amzad Hossain M Shah MD. A study on the total phenols content and antioxidant activity of essential oil and different solvent extracts of endemic plant Merremia borneensis. Arab J Chem. (2015) 8:66–71. 10.1016/j.arabjc.2011.01.007

[69] Aryal S Baniya MK Danekhu K Kunwar P Gurung R Koirala N. Total phenolic content, Flavonoid content and antioxidant potential of wild vegetables from western Nepal. Plants. (2019) 8:96. 10.3390/plants8040096 30978964 PMC6524357

[70] Alam MK Rana ZH Kabir N Begum P Kawsar M Khatun M. Total phenolics, total carotenoids and antioxidant activity of selected unconventional vegetables growing in Bangladesh. Curr Nutr Food Sci. (2019) 16:1088–97. 10.2174/1573401315666191209095515

[71] Lu Q Huang N Peng Y Zhu C Pan S. Peel oils from three Citrus species: volatile constituents, antioxidant activities and related contributions of individual components. J Food Sci Technol. (2019) 56:4492–502. 10.1007/s13197-019-03937-w 31686681 PMC6801276

